# Relationship of possible biomarkers with malignancy of thymic tumors: a meta-analysis

**DOI:** 10.1186/s12885-020-07332-z

**Published:** 2020-09-29

**Authors:** Huilan Zeng, Weilin Yang, Bo Xu, Jianyong Zou, Chunhua Su, Beilong Zhong, Haoshuai Zhu, Zhenguang Chen

**Affiliations:** 1 Department of Thoracic Surgery and Department of Cardiothoracic Surgery of East Division, the First Affiliated Hospital of Sun Yat-Sen University, Guangzhou, No. 58, Zhongshan Road II, Guangzhou, Guangdong 510080 P. R. China; 2 Department of Thoracic Surgery, the First Affiliated Hospital of Sun Yat-Sen University, Guangzhou, Guangdong 510080 P. R. China; 3 Department of Thoracic Surgery, the Fifth Affiliated Hospital of Sun Yat-Sen University, Zhuhai, Guangdong 519000 P. R. China

**Keywords:** Biomarkers, Proliferation, Apoptosis, Thymic malignant tumors, Malignancy

## Abstract

**Background:**

Role of biomarkers for promotion of tumor proliferation (BPTPs) and for promotion of apoptosis (BPAs) in thymic malignant tumors is still unclear. The purpose of this study was to evaluate the relationship between BPTPs and/or BPAs and malignancy of thymic malignant tumors.

**Methods:**

Studies on thymic malignant tumors and biomarkers were searched in PubMed, ISI Web of Knowledge, and Embase databases, and all statistical analyses were conducted using Review Manager.

**Results:**

Twelve articles related to biomarkers and thymic malignant tumors were selected and analyzed. A relationship between BPAs and Masaoka stage was demonstrated for four markers, namely Bax, p73, Casp-9 and Bcl-2, included 138 stage I/II patients and 74 stage III/IV patients, and BPAs were significantly correlated with high Masaoka staging (*P* = 0.03). We further found a relationship between BPAs and degree of malignancy for four markers, namely Bax, p73, Casp-9 and Bcl-2, included 176 thymoma patients and 36 thymic carcinoma patients, and BPAs were significantly correlated with thymic carcinoma (*P* = 0.010). In addition, a relationship between BPTP and Masaoka staging was demonstrated for seven markers, namely Podoplanin, Glut-1, Muc-1, Egfr, Igf1r, c-Jun, and n-Ras, included 373 patients with stage I/II and 212 patients with stage III/IV, and BPTPs were significantly correlated with high Masaoka staging (*P* < 0.001). We also found a relationship between BPTPs and degree of malignancy for ten markers, namely Mesothelin, c-Kit (CD117), Egfr, Lat-1, Muc-1,Ema, Glut-1, Igf1r, c-Jun, and n-Ras, included 748 thymoma patients and 280 thymic carcinoma patients, and BPTPs were significantly correlated with thymic carcinoma (P < 0.001).

**Conclusion:**

These findings show that high levels of BPTPs or BPAs are more closely related to thymic carcinoma and Masaoka stage III/IV, suggesting that BPTPs and BPAs may play an important role in the occurrence and development of thymic malignant tumors.

## Background

Thymic malignant tumors, including malignant thymoma and thymic carcinoma, are a group of rare diseases with unknown etiology that are often diagnosed based on histological criteria. Both of these malignancies, especially thymic carcinoma, show cytologic atypia, invasive margins, and loss of an organotypic appearance [1]. It is generally considered that the biological characteristics of malignant thymoma and thymic carcinoma are related to their staging and pathological malignancy, and that the prognosis of surgical resection for malignant thymoma and thymic carcinoma is worse than that for thymoma in the early phase [1–7]. Unfortunately, because of a lack of uniform measurement standards, determining the prognosis of malignant thymoma and thymic carcinoma based on an analysis of histological type is complicated. One of the main features of the present system for classifying thymomas, as exemplified by the World Health Organization (WHO) classification system, is the proportion of lymphocytes in the thymus tumor. According to this system, WHO type A and type B1 are believed to be less invasive, whereas type B2 and type B3 are considered to be more invasive [8–11].

Recent studies have explored the relationship between the expression of several biomarkers and the prognosis or diagnosis of malignancy in thymic malignant tumors. These markers include EGFR (epidermal growth factor receptor), GLUT1 (glucose transporter 1), EMA (epithelial membrane antigen), IGF-1R (insulin-like growth factor I receptor), BAX (BCL2 associated X), p73, BCL2 (B cell leukemia/lymphoma 2), PD-L1 (programmed death ligand 1), FOXP3 (forkhead box P3) and TdT (terminal deoxynucleotidyl transferase), among others [12–21]. In addition, increased expression of tumor-associated genes, such as FPGS (folylpolyglutamate synthase)/GGH (gamma-glutamyl hydrolase) and VEGF (vascular endothelial growth factor), were found to be related to the degree of malignancy in thymic carcinoma and B3 thymoma [8]. Notably, C-kit expression, which is detectable in approximately 70–86% of patients with thymic carcinoma, is only found in 0–5% of thymic adenomas [10, 22]. The epigenetics of thymoma genes has also been investigated, including histone modification, chromatin recombination, and gene methylation [11, 22]. However, although such studies have identified a number of biomarkers related to thymic malignant tumors, they have often reported quite different or even completely opposite results and have sampled limited population sizes [16, 22–28]. Because of these limiting factors, there is no definitive conclusion as to which markers are capable of reflecting the degree of malignancy or Masaoka stage of thymomas.

On the other hand, some biomarkers, such as EGFR, GLUT-1 and IGF-1R, have similar characteristics that impact the proliferative potential and invasive ability of tumors. Mutation of the apoptosis-related proteins, BCL-2, p53 or BAX, can lead to the occurrence and development of thymic malignant tumors. Expression of FOXP3 and TdT in cancer cells suggests that T cells may play a part in tumor immune escape. Given their similar characteristics, Podoplanin, Muc-1, Glut-1, Egfr,Igf1r, c-Jun, n-Ras, Mesothelin, c-Kit (cd117), Lat1, and Ema, have been classified as biomarkers for promotion of tumor proliferation (BPTPs); Bax, p73, Casp-9 and Bcl-2 have been classified as biomarkers for promotion of tumor apoptosis (BPAs); and FOXP3 and TdT have been classified as T cell markers. Given the relative lack of literature on the relationship of T cell markers, B cell markers and mitogenic markers with thymic malignant tumors, studies on the association of BPAs or BPTPs with thymic malignant tumors are particularly noteworthy.

Strong expression of BPAs is associated with advanced thymoma and thymic carcinoma [13]. Studies have shown that thymic malignant tumors expressing BPTPs tend to be advanced and highly malignant [12, 14, 17, 18, 23, 29–33]. However, this conclusion is tempered by the small sample sizes involved and discrepancies among reports. For example, the frequency of BPA or BPTP positivity in Masaoka stage III/IV was showed in higher level than that in stage I/II, but it was also found no statistically significant difference in BPA or BPTP positivity according to Masaoka stages [13] Similarly, the frequency of BPA or BPTP positivity in thymic carcinoma was showed higher level than that in thymoma, but other studies found no significant difference between these tumor types [12, 13, 34]. Along the same lines, it suggested that the rate of apoptosis in thymoma is higher than that in thymic carcinoma [22].

Given the high expression of numerous molecular markers in a variety of cancers and their association with poor prognosis, as well as controversies surrounding the significance of their expression in thymic epithelial tumors, we conducted this study to determine whether BPTPs and/or BPAs contribute to the staging and degree of malignancy of thymic malignant tumors.

## Methods

### Search strategy

A comprehensive literature review in PubMed, ISI Web of Knowledge, and Embase databases was conducted using the key words “markers”, “thymoma”, “thymic adenocarcinoma” and “thymic malignant tumor”. Articles published as of December 30, 2018, were collected, including case-control and cohort studies on thymoma and tumor markers.

### Inclusion criteria

The following criteria were used to select documents for further meta-analysis: (1) published in English, regardless of the publication time; (2) evaluated the relationship between BPTPs (e.g., EGFR, GLUT-1, IGF-1R) or BPAs (e.g., Bcl-2, p73, Bax) and cancer stage or degree of malignancy; (3) confirmed cancer patients by pathology; (4) including detailed cancer/Masaoka staging data; and (5) divided thymic malignant tumor patients into at least two groups, namely, thymic adenocarcinoma versus thymic carcinoma or Masaoka stage I/II versus stage III/IV.

### Data extraction

Two researchers independently reviewed each article under the guidance of an instructor from the same center. Details of publication characteristics for each qualified publication, including the first author’s name, year of publication, patient’s country of origin and race, total number of patients, cancer type, and median/average age and disease stage of the study population, were collected. Positive or high levels of markers and Masaoka staging or degree of malignancy were the focus of attention.

### Statistical analysis

Four different relationships were analyzed: (1) the relationship between BPAs and Masaoka stage; (2) the relationship between BPAs and thymoma malignancy; (3) the relationship between BPTPs and Masaoka stage; and (4) the relationship between BPTPs and thymoma malignancy. Correlations between marker positivity/high expression and degree of malignancy and stage of thymic malignant tumors were determined by measuring odds ratios (ORs) and relevant 95% confidence intervals (CIs). Statistical heterogeneity between studies was evaluated using Cochran’s heterogeneity statistics Q and I2, which describe the variation caused by heterogeneity rather than random error, as follows: I2 = 0–25%, no heterogeneity; I2 = 25–50%, moderate heterogeneity; I2 = 50–75%, large heterogeneity; and I2 = 75–100%, extreme heterogeneity. In the initial analysis, a fixed effects model was applied; a confirmed random effects model was used in cases where there was significant heterogeneity. A funnel chart was used to evaluate publication deviation. All statistical analyses were conducted using Review Manager Version 5.0 (RevMan Cochrane Collaboration, Oxford, UK). All *P*-values in meta-analysis were bilateral, and *P*-values less than 0.05 were considered significant.

## Results

### Research characteristics

**Fig. 1:**
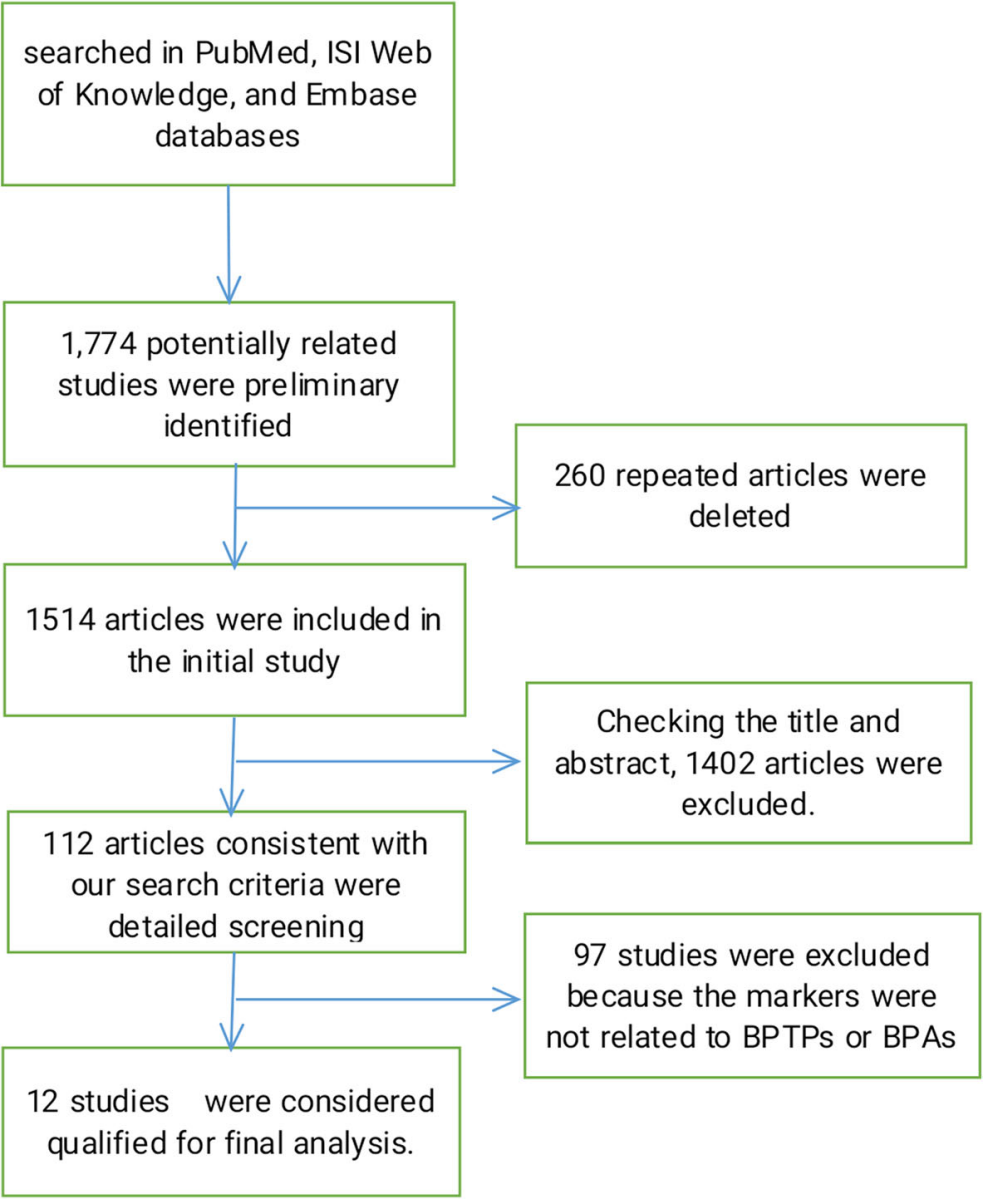
PRISMA flow-diagram

Our initial search strategy identified 1774 potentially related studies. 260 repeated articles were deleted and the remaining 1514 articles were included in the initial study. After reading the title and abstract, we selected 112 articles that met our search criteria for further detailed evaluation. After careful screening, 97 studies were excluded because the markers in these studies were not related to tumor proliferation or apoptosis. Of the remaining 15 studies, three did not have sufficient data. In the end, 12 studies of markers and degree of malignancy or tumor stage were considered qualified for final analysis. The characteristics of the included studies are listed in Tables 1, 2, 3, 4. PRISMA flow-diagram was shown in Fig. 1.

**Table 1:** Characteristics of the studies included in the meta-analysis: relationship between BPAs and Masaoka staging

First author	Year	Country	Ethnicity	No. of patients	Median age (years)
Kenzo Hiroshima [16]	2002	Japan	Japanese	46	56.3
Yuqing Ma [17]	2012	China	Chinese	60	48.5

**Table 2:** Characteristics of the studies included in the meta-analysis: relationship between BPAs and degree of malignancy

First author	Year	Country	Ethnicity	No. of patients	Median age (years)
Kenzo Hiroshima [16]	2002	Japan	Japanese	46	56.3
Yuqing Ma [17]	2012	China	Chinese	60	48.5

**Table 3:** Characteristics of the studies included in the meta-analysis: relationship between BPTPs and Masaoka staging

First author	Year	Country	Ethnicity	No. of patients	Median age (years)
T. Mimae [12]	2012	Japan	Japanese	140	54
Hisashi Tateyama [30]	2011	Japan	Japanese	99	54
Yuqing Ma [17]	2012	China	Chinese	60	48.5
Jun Du [13]	2016	China	Chinese	43	51

**Table 4:** Characteristics of the studies included in the meta-analysis: relationship between BPTPs and degree of malignancy

First author	Year	Country	Ethnicity	No. of patients	Median age (years)
Lchiro Fukai [14]	1992	Japan	Japanese	95	NR
T. Mimae [12]	2012	Japan	Japanese	140	54
Kiyotaka Yoh [16]	2008	Japan	Japanese	38	61
Kyoichi Kaira [32]	2011	Japan	Japanese	55	61
Kyoichi Kaira [33]	2009	Japan	Japanese	45	55
Chinchen Pan [35]	2004	China	Chinese	132	NR
Anish Thomas [36]	2016	America	American	71	51
Yuqing Ma [17]	2012	China	Chinese	60	48.5
Daisuke Nonaka [23]	2007	America	American	75	59
Jun Du [13]	2016	China	Chinese	43	51

### Publication bias

Funnel charts of the four groups are shown in the Figure. No obvious asymmetry was detectable in any of the four groups, demonstrating the absence of publication bias.

### Relationship between BPAs and Masaoka staging

**Fig. 2:**
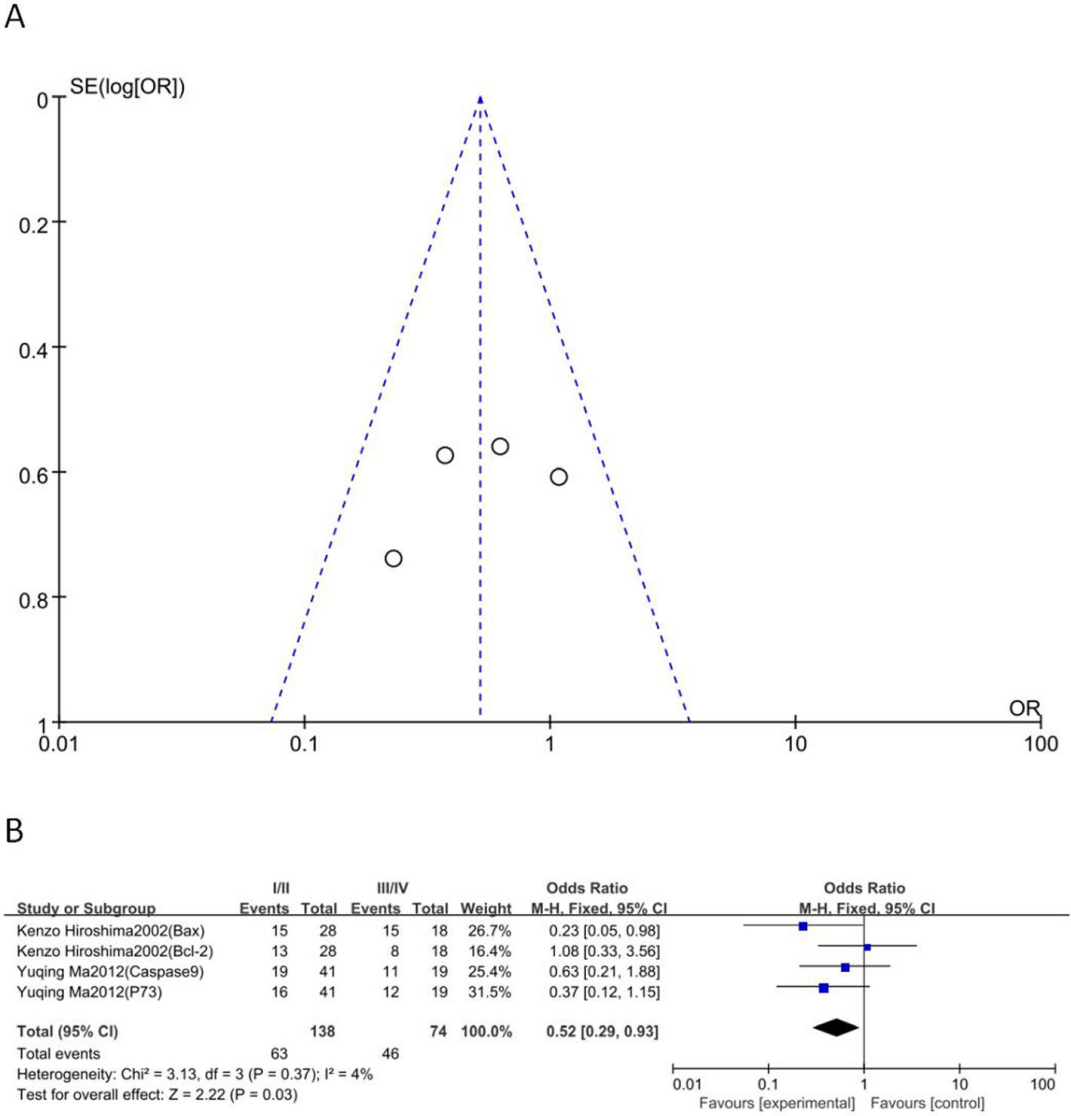
Relationship between BPAs and Masaoka staging. a, Funnel plot. **b**, Forest plot

Four markers, namely Bax, p73, Casp-9 and Bcl-2, from two articles that included 138 patients in phase I/II and 74 patients in phase III/IV were selected. Combining the results from these two eligible studies in a meta-analysis revealed evidence of a correlation between positive/highly expressed pro-apoptotic tumor markers and thymoma stage III/IV. As shown in Fig. 2b, significant major effects were observed between positive/highly expressed BPAs and Masaoka stage III/IV (I/II vs. III/IV: OR 0.52, 95% CI 0.29–0.93; *P* = 0.03).

### Relationship between BPAs and degree of malignancy

**Fig. 3:**
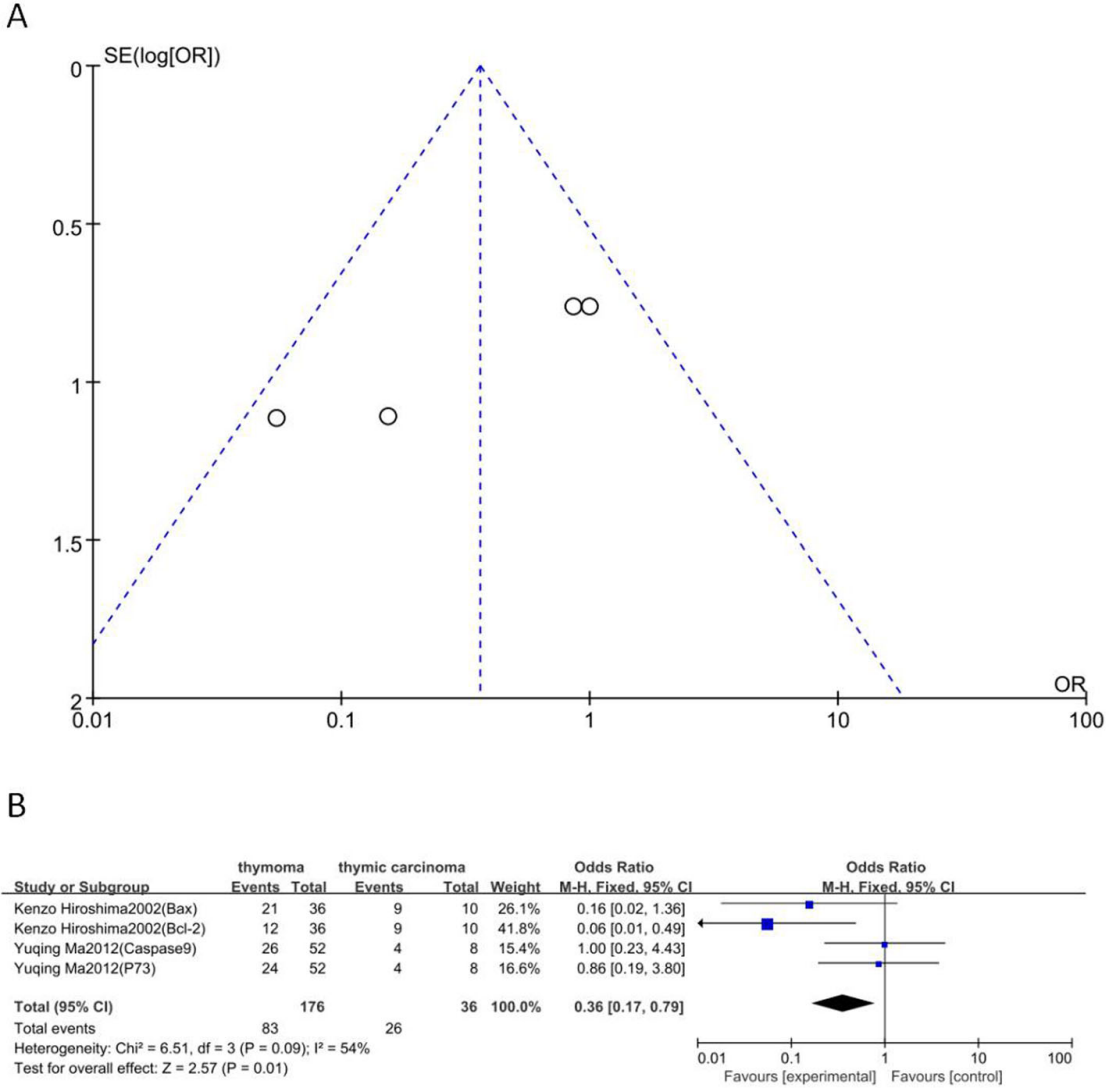
Relationship between BPAs and degree of malignancy. **a**, Funnel plot. **b**, Forest plot

Four markers, namely Bax, p73, Casp-9 and Bcl-2, from two articles that included 176 cases of thymoma and 36 cases of thymic carcinoma were selected. As shown in Fig. 3b, significant major effects were observed between positive/highly expressed BPAs and thymic carcinoma (thymoma vs. thymic carcinoma: OR 0.36, 95% CI 0.17–0.79; *P* = 0.01).

### Relationship between BPTP and Masaoka staging

**Fig. 4:**
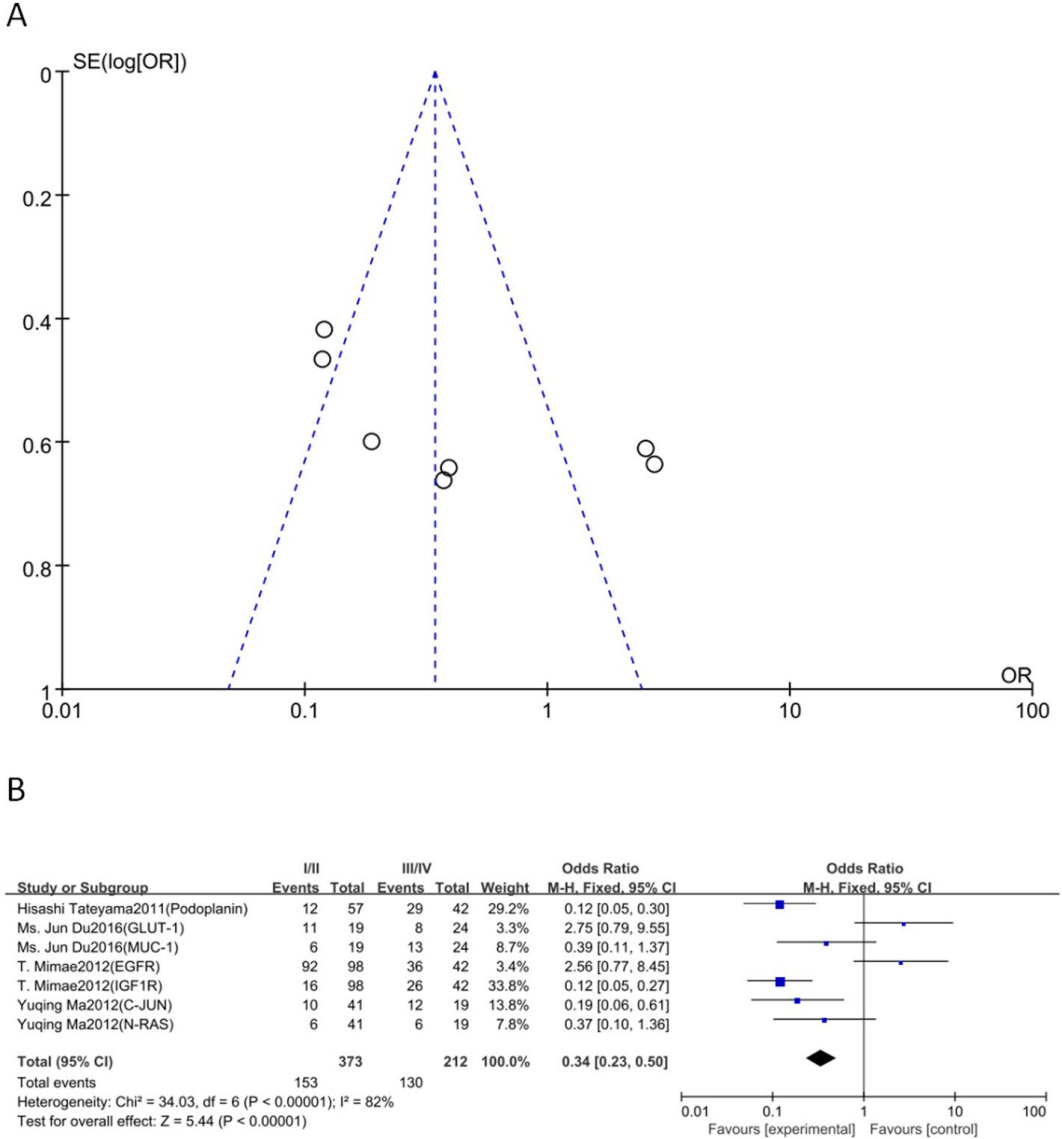
Relationship between BPTPs and Masaoka staging. **a**, Funnel plot. **b**, Forest plot

Seven markers, namely Podoplanin, Glut-1, Muc-1, Egfr, Igf1r, c-Jun, and n-Ras, from five articles that included 373 patients in phase I/II and 212 patients in phase III/IV were selected. As shown in Fig. 4b, significant major effects were observed between positive/highly expressed BPTPs and Masaoka stage III/IV (I/II vs. III/IV: OR 0.34, 95% CI 0.23–0.50; *P* < 0.00001).

### Relationship between BPTPs and degree of malignancy

**Fig. 5:**
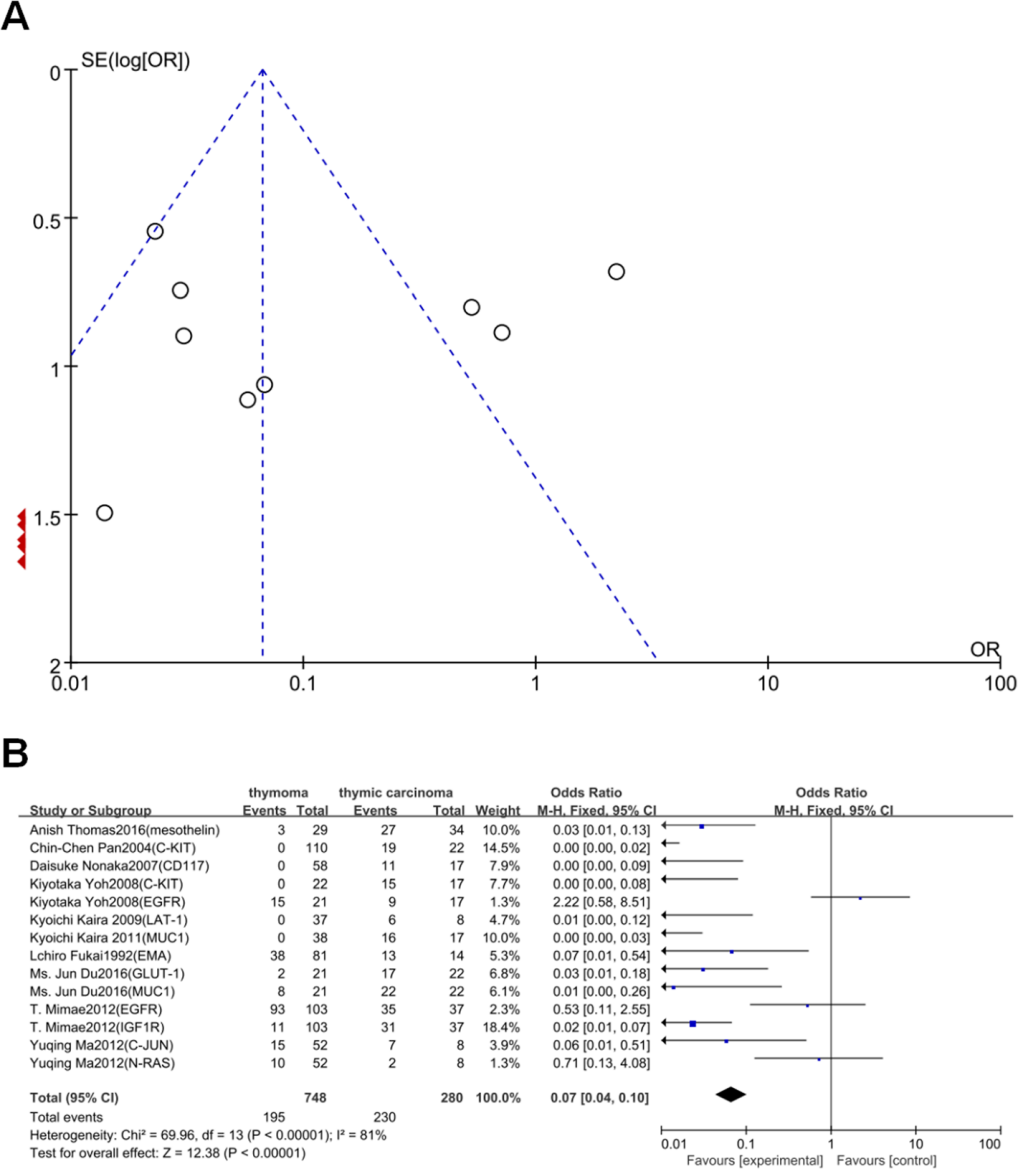
Relationship between BPTPs and degree of malignancy. **a**, Funnel plot. **b**, Forest plot

Ten markers, namely Podoplanin, Glut-1, Muc-1, Egfr, Igf1r, c-Jun, and n-Ras, from ten articles that included 748 cases of thymoma and 280 cases of thymic carcinoma were selected. As shown in Fig. 5b, significant major effects were observed between positive/highly expressed BPTPs and thymic carcinoma (thymoma vs. thymic carcinoma: OR 0.07, 95% CI 0.04–0.10; *P* < 0.00001).

### Heterogeneity test

Using Q statistics and applying a random-effects model, we observed statistically significant heterogeneity between the following tests: BPAs and thymoma versus thymic carcinoma (*P* = 0.09, I2 = 54%); BPTPs and phase I/II versus phase III/IV (*P* < 0.00001, I2 = 82%); and BPTPs and thymoma versus thymic carcinoma (*P* < 0.00001, I2 = 85%). We found no obvious heterogeneity between BPAs and Masaoka stage (*P* = 0.75, I2 = 0%); therefore, a fixed effect model was used for this analysis.

## Discussion

An excess of bax protein promotes apoptosis. Proteins of the B-cell lymphoma-2 (BCL-2) family control the intrinsic apoptosis pathway. The pro-apoptotic BCL-2 proteins BAX and BAK can commit a cell to its programmed death by permeabilizing the outer mitochondrial membrane (OMM) and subsequent initiation of the caspase cascade [16, 37].

Apoptosis or programmed cell death is a fundamental cellular process that is paramount for cellular regeneration and tissue homeostasis in multicellular organisms. Unlike other cell death pathways, apoptosis efficiently dismantles the cell without adverse effects on neighboring cells or its environment. Its faithful execution is essential in avoiding a number of catastrophic disease states and is also critical in organismal development, so apoptosis is very tightly regulated. Caspases, aspartate-directed, cysteine proteases play prominent roles in apoptotic pathways. Initiator caspases (caspase-2, − 8 and − 9) function upstream of the apoptotic pathways while executioners (caspase-3, − 6 and − 7) mediate downstream reactions [35]. As part of the p53 family, with different kinds of promoter transcription and alternative splicing, p73 can produce > 10 different subtypes, collectively called DNp73 or ΔTAp73, that play an important role in the expression of human tumors. The DNp73 is different from p53 in function due to the significant difference in their structures, while the ΔTAp73 has similar functions as p53 in inhibiting tumor and promoting apoptosis [36].

Given their similar characteristics, Bax, p73, Casp-9 and Bcl-2 have been classified as biomarkers for promotion of tumor apoptosis (BPAs) in this study.

In head and neck squamous cell carcinoma, overexpression of podoplanin is associated with lymph node metastasis and poor clinical outcome. MUC1 oncoprotein is aberrantly expressed at high levels in most human neoplasms, and MUC1 plays important roles in development and progression of malignant tumors. Several studies have identified GLUT1 as a prognostic and diagnostic marker and it has been found to be associated with tumor progression and poor overall survival in various malignant tumors [18, 29].

Insulin-like growth factor-1 receptor (IGF-1R), epidermal growth factor receptor (EGFR), human epidermal growth factor receptor-type 2 (HER2), and c-Met are members of the receptor tyrosine kinases (RTKs). Receptor tyrosine kinases (RTKs) have been shown to have critical roles in the proliferation, migration, and survival of many types of malignant neoplastic cells. C-Jun can directly act as an oncogene by helping in the proliferation and consequent invasion and metastasis of the cells. Zheng ZY found that wild-type N-RAS is overexpressed in BLBCs. Repressing N-RAS inhibits transformation and tumor growth, whereas overexpression enhances these processes even in preinvasive BLBC cells. Mesothelin is a cell-surface antigen implicated in tumor invasion, which is highly expressed in mesothelioma, lung, pancreas, breast, ovarian, and other cancers [12, 38, 39].

CD117 is involved in the development of several malignant tumor types including gastrointestinal stromal cell tumors, small-cell lung, ovarian and breast cancer. Immunohistochemical staining has revealed that CD117 protein is overexpressed in primary malignant tumors, including operable esophageal squamous cell carcinoma and vulvar melanoma, and may be a valuable prognostic marker in esophageal squamous cell carcinoma. L-type amino acid transporter 1(Lat-1) is one of the amino acid transporters, which are necessary for tumor growth and proliferation, and is highly expressed in many cancer cells. In addition, LAT1 was highly expressed in patients with pancreatic cancer, and its expression yielded a significant association with cell proliferation, angiogenesis, and disease stage. Sloane and Ormerod reported that EMA staining of various tumors is related to the degree of tumor differentiation. The increase of EMA expression after malignant neoplastic transformation is suggested to be related to poor intercellular contact, which may help to sustain the unrestricted growth characteristic of neoplasms [14, 40, 41].

Given their similar characteristics, Bax, p73, Casp-9 and Bcl-2 have been classified as biomarkers for promotion of tumor apoptosis (BPAs); Podoplanin, Muc-1, Glut-1, Egfr,Igf1r, c-Jun, n-Ras, Mesothelin, c-Kit (cd117), Lat1, and Ema, have been classified as biomarkers for promotion of tumor proliferation (BPTPs).

BPAs and/or BPTPs are often used to predict the occurrence and development of malignancy and monitor effects of therapy on tumors. Such biomarkers have been widely used in the diagnosis and treatment of malignant tumors. Examples include treatment of EGFR-mutation–positive lung cancer with mutant-EGFR–targeted inhibitors, or elevation of AFP as an indicator of liver cancer. However, the mechanisms underlying the involvement of BPAs and BPTPs in the development of thymic malignant tumors, especially their relationship to clinical stage or pathological malignancy, have remained unclear. In the present study, we systematically identified and evaluated existing data, and analyzed the relationships between the rate of tumor marker positivity, reflecting tumor proliferation and apoptosis, and thymoma versus cancer and phase I/II versus phase III /IV. These analyses revealed evidence of a significant association of BPAs or BPTPs with thymic adenocarcinoma or stage III/IV thymic malignant tumors.

Our demonstration of a significant relationship between positivity/high expression of BPAs or BPTPs and thymic adenocarcinoma or stage III/IV thymic malignant tumors suggests that gene products that promote tumor proliferation or apoptosis may play an important role in the occurrence and development of thymic malignant tumors. However, further investigation of thymic malignant tumors is needed to confirm our results. We will continue to do further research. Analyse patient’s sample with Histo-pathological technique and real time PCR using BPTPS and BPA markers, Using thymoma patient specimens for total gene detection. If the results of both of them also indicate that the staging and malignancy of thymoma are related to BPTPs or BPAs, our conclusion will be more effectively verified.

## Conclusions

Collectively, our findings provide evidence of a correlation between expression of BPAs or BPTPs and Masaoka staging or pathological malignancy of thymoma. Therefore, high levels or positive expression of BPAs or BPTPs may be strongly linked to high Masaoka stages of thymoma or more prominent pathological malignancy.

## Data Availability

Data sharing not applicable to this article as no datasets were generated or analyzed during the current study.

## Abbreviations

BPTPs: Biomarkers for promotion of tumor proliferation
BPAs: Biomarkers for promotion of apoptosis
GLUT-1: Glucose transporter 1
EGFR: Epidermal growth factor receptor
IGF1R: Insulin-like growth factor-1 receptor
LAT1: L-type amino acid transporter 1
EMA: Epithelial membrane antigen
